# Major disparities in COVID-19 test positivity for patients with non-English preferred language even after accounting for race and social factors in the United States in 2020

**DOI:** 10.1186/s12889-021-12171-z

**Published:** 2021-11-18

**Authors:** Hannah Cohen-Cline, Hsin-Fang Li, Monique Gill, Fatima Rodriguez, Tina Hernandez-Boussard, Harry Wolberg, Jacob Lippa, Keri Vartanian

**Affiliations:** 1 Center for Outcomes Research and Education, Providence St. Joseph Health, 5251 NE Glisan St., Portland, OR 97213 USA; 2 Cardiovascular Analytics, Research, and Data Science, Providence St. Joseph Health, 9205 SW Barnes Road, Portland, OR 97225 USA; 3 Division of Cardiovascular Medicine and the Cardiovascular Institute, Stanford University, 291 Campus Drive, Stanford, CA 94305 USA; 4 Department of Medicine (Biomedical Informatics), Stanford University, 291 Campus Drive, Stanford, CA 94305 USA; 5 Clinical Analytics, Providence St. Joseph Health, 1801 Lind Ave SW, Renton, WA 98057 USA

**Keywords:** COVID-19 disparity, Preferred language, Race/ethnicity, Social factors

## Abstract

**Background:**

The COVID-19 pandemic has further exposed inequities in our society, demonstrated by disproportionate COVID-19 infection rate and mortality in communities of color and low-income communities. One key area of inequity that has yet to be explored is disparities based on preferred language.

**Methods:**

We conducted a retrospective cohort study of 164,368 adults tested for COVID-19 in a large healthcare system across Washington, Oregon, and California from March – July 2020. Using electronic health records, we constructed multi-level models that estimated the odds of testing positive for COVID-19 by preferred language, adjusting for age, race/ethnicity, and social factors. We further investigated interaction between preferred language and both race/ethnicity and state. Analysis was performed from October–December 2020.

**Results:**

Those whose preferred language was not English had higher odds of having a COVID-19 positive test (OR 3.07, *p* < 0.001); this association remained significant after adjusting for age, race/ethnicity, and social factors. We found significant interaction between language and race/ethnicity and language and state, but the odds of COVID-19 test positivity remained greater for those whose preferred language was not English compared to those whose preferred language was English within each race/ethnicity and state.

**Conclusions:**

People whose preferred language is not English are at greater risk of testing positive for COVID-19 regardless of age, race/ethnicity, geography, or social factors – demonstrating a significant inequity. Research demonstrates that our public health and healthcare systems are centered on English speakers, creating structural and systemic barriers to health. Addressing these barriers are long overdue and urgent for COVID-19 prevention.

## Background

Disparities in health care and health outcomes by race, ethnicity, and socioeconomic status have been documented for decades [1, 2]. Like past pandemics in our history, the COVID-19 pandemic has further exposed these inequities as seen by the disproportionate COVID-19 infection rate and mortality in communities of color and communities facing socioeconomic barriers [3–5]. These disparities emerged in the very early days of the pandemic and continue to be documented [3–7].

People of color and those with low socioeconomic status are also more likely to face language barriers when engaging in the healthcare system [8], which occur when there is a difference in native language between patients and providers and/or systems. In the United State (U.S.), because the healthcare system predominantly serves English-speakers, this most commonly occurs when English is not the native or preferred language of the patient. Language barriers are a well-established driver of inequitable outcomes in health care [9, 10], but less is known about the role of language barriers in COVID-19 infection and outcomes.

In the U.S., approximately 25 million people (8.4% of the population) report that they speak English “less than very well.” [11] The size of the population varies dramatically by state; for example, across the West Coast, the percent in California climbs to 17.8%, while Oregon and Washington state have lower percentages at 5.6 and 7.6%, respectively [11]. Research demonstrates that those whose preferred language is not English have worse health and health care outcomes, including decreased access to health insurance, longer lengths of stay in the hospital, greater readmission rates, worse management of chronic disease, greater risk of having adverse medical events, and lower overall satisfaction with their care [9, 10, 12–16]. These worse outcomes often arise from worse patient experience including unmet informational needs leading patients to be uninformed about their health, lack of cultural safety, and discrimination [15]. Further, the population whose preferred language is not English, many of whom are immigrants, are also at greater likelihood of having social factors associated with COVID-19 infection such as living in multigenerational households, being “essential workers” who are unable to work from home, relying on public transportation, and having lower socioeconomic status [4, 17–19]. Taken together, these experiences represent a potentially synergistic myriad of factors that could increase risk for COVID-19 infection and worsened outcomes.

Some studies have begun to explore the importance of language in COVID-19 risk and care. One study created clinical and social risk models for COVID-19 infection and found that non-English speaking patients had 2.09 greater odds of COVID-19 infection compared to English-speaking patients [4]. Similarly, another study of COVID-19 patients found that the non-English speaking population had significantly increased risk of hospitalization within the first 45 days of diagnosis compared to English speakers; this finding was independent of an area deprivation index and was consistent across race/ethnicity [6]. Another study out of Massachusetts General Hospital showed that their Spanish-speaking patient population with limited English-proficiency increased nearly 20 times during the pandemic - ultimately making up more than 40% of their COVID-19 infected patients [19]. Despite this early evidence, there have been no comprehensive studies focused on understanding the role of language barriers in COVID-19 infection risk nor has anyone explored the complex intersection of language, race, and social factors in this context.

In this study, we used electronic health records (EHR) from a large hospital system spanning multiple states to examine COVID-19 test positivity among those who identified English as a preferred language compared to those whose preferred language was not English. We aimed to determine whether preferred language led to an increased likelihood of having a positive COVID-19 test independent of race/ethnicity, social factors, and geography. This study provides new information about the impact of having a preferred language that is not English in the U.S. on COVID-19 positivity and is particularly important as clear action could be taken to address the structural barriers that exist within and outside of the U. S public health and healthcare systems for people whose preferred language is a language other than English.

## Methods

### Setting and population

This retrospective cohort study was conducted at Providence St. Joseph Health (PSJH), − the third largest multi-state healthcare system in the country, and the first to treat a known COVID-19 case in the U.S. Analyses were restricted to adult patients who were tested for COVID-19 at a PSJH facility in Washington, Oregon, or California between March and July 2020. Our data source for identifying PSJH patients tested for COVID-19 was EHR; our analysis was limited to hospitals using the same instance of EHR. The protocol for this study was approved by the PSJH Institutional Review Board (IRB #2020000494).

### Data collection

Data for this study were obtained from PSJH’s EHR system. We identified patients who received a PCR or antigen test for COVID-19 at a PSJH facility during the study period and were at least 18 years old. Duplicate encounters were removed and patients without test results or with encounters outside of Washington, Oregon, and California were excluded, resulting in a final sample size of 164,368 patients.

Patient demographic information was extracted from the EHR, including age, sex, language, self-reported race/ethnicity, state of residence, and payer type. Self-reported race/ethnicity categories were mutually exclusive and had the following hierarchical order: 1. Hispanic (all races), 2. Multiracial, 3. Single primary race. The preferred language listed on the EHR was used to classify patients as English-speakers or individuals whose preferred language was not English. In the EHR, the preferred a language that was not English category included over 100 languages, with Spanish, Vietnamese, Armenian, and Russian being the most common languages after English. This selection may have been made by the patient or provider.

For the encounter associated with the COVID-19 test, payer type (commercial, Medicaid, Medicare, other, or self-pay) and setting (emergency department, inpatient, outpatient, virtual, or other) were also assessed from the EHR. Patients were designated as symptomatic at testing if the EHR reported any of the following symptoms: cough, shortness of breath, fever, sore throat, rhinorrhea, nausea or vomiting, headache, myalgia, chills, or wheezing. Date of COVID-19 test and test results were also extracted from the EHR.

Social factors included in this study were identified at a census-track level using the Social Vulnerability Index (SVI). Patient address in the EHR was used to identify census tract of residence, which was then matched to their SVI. The SVI uses census data to estimate the relative vulnerability of each census tract in the U.S. by ranking 15 variables in 4 major themes: socioeconomic status, household composition and disability, minority status and language, and housing and transportation [20]. This analysis used the socioeconomic status and housing/transportation domains as they were hypothesized to be the most relevant to COVID-19 infection risk because of the increased risk of exposure to other individuals due to crowded living situations, public transportation needs, or type of employment. Scores range from 0 to 1 for each domain, with a score closer to 1 representing greater vulnerability.

### Statistical analyses

We first explored the trends and disparities in our data to support building models that would answer our research question [21, 22]. Sociodemographic characteristics of the sample were summarized using descriptive statistics, stratified by preferred language. Using the date of testing and test results, rates of COVID-19 test positivity were also explored over time by preferred language.

Mixed effect logistic regression models were used to assess factors associated with test positivity. First, an unadjusted univariate multilevel model was calculated using language as an independent variable (Model A). Next, a series of adjusted multivariate multilevel models were built to incrementally include age and race/ethnicity (Model B), and SVI estimates (Model C) as covariates. Covariates were selected a priori based on our research questions of interest and previous literature rather than statistical model selection techniques. Lastly, to explore intersectionality between preferred language and race and preferred language and geography, interactions between language and race/ethnicity (Model D) and language and state (Model E) were tested. For the interaction between language and race/ethnicity, we excluded the American Indian/Alaskan Native group due to the very small sample size concerns. State was included as a nested random effect in all models to account for geographical variation in COVID-19 spread and testing availability. Odds ratios (ORs) and 95% confidence intervals (CIs) were generated for all models. All analyses were performed in October–December 2020 using SAS Enterprise Guide 7.1 (SAS Institute Inc., Cary, NC, USA.).

## Results

A total of 164,368 tested for COVID-19 were included in analyses, including 150,198 English-speakers and 14,170 people who preferred a language that was not English (Table 1).

**Table 1:** Study Population Demographics, Healthcare, and, SVI in the Western U.S. in 2020

	Preferred English***N*** = 150,198		Preferred language that is not English***N*** = 14,170	
	N	%	N	%
Age^a^ (years)	51.4	19.5	56.1	19.5
Sex				
Female	85,674	57.1%	7840	55.4%
Male	64,494	42.9%	6316	44.6%
Race & Ethnicity				
American Indian/Alaska Native	1010	0.7%	6	0.0%
Asian	5637	3.8%	1526	10.8%
Black/African American	5916	3.9%	186	1.3%
Hispanic	14,622	9.7%	6318	44.6%
Multiracial	3596	2.4%	171	1.2%
Native Hawaiian/Pacific Islander	833	0.6%	117	0.8%
Other	4770	3.2%	859	6.1%
White	101,535	67.6%	1359	9.6%
*Missing*^*b*^	*12,279*	*8.2%*	*3628*	*25.6%*
State				
California	41,415	27.6%	7139	50.4%
Oregon	35,780	23.8%	3289	23.2%
Washington	73,003	48.6%	3742	26.4%
Symptomatic^c^ at testing	81,085	54.0%	7622	53.8%
Payer Type				
Commercial	56,168	37.4%	2812	19.8%
Medicaid	19,585	13.0%	2865	20.2%
Medicare	37,094	24.7%	3712	26.2%
Other Insurance	4592	3.1%	574	4.1%
Self-Pay	32,759	21.8%	4207	29.7%
Encounter Setting				
Emergency Department	36,478	24.3%	3078	21.7%
Inpatient	31,109	20.7%	4318	30.5%
Other	47,263	31.5%	3759	26.5%
Outpatient	8463	5.6%	618	4.4%
Virtual	26,885	17.9%	2397	16.9%
SVI Domain: Socioeconomic^a^	0.40	0.26	0.53	0.29
SVI Domain: Housing and Transportation^a^	0.56	0.29	0.62	0.30

^a^mean and standard deviation

^b^includes declined/unknown

^c^defined as reporting any of the following symptoms: cough, shortness of breath, fever, sore throat, rhinorrhea, nausea or vomiting, headache, myalgia, chills, wheezing

Among English-speaking patients, the average age was 51.4 years, and 57.1% were female. Over two-thirds (67.6%) of English-speaking patients identified as White and 8.2% were missing race/ethnicity in the EHR. Roughly half (48.6%) of patients in this group were seen in Washington, with 23.8% in Oregon and 27.6% in California. English-speaking patients primarily had commercial payers (37.4%), Medicare (24.7%), or self-pay (21.8%), and encounters primarily took place in “other” settings (31.5%), EDs (24.3%), or inpatient settings (20.7%). The communities where English-speaking patients lived scored an average of 0.40 and 0.56 on the SVI Socioeconomic and SVI Housing and Transportation domains, respectively.

Among patients whose preferred language was not English, the average age was 56.1 years, and females accounted for 55.4% of this group. Nearly half (44.6%) of patients whose preferred language was not English identified as Hispanic, 10.8% identified as Asian, 9.6% identified as White, and 25.6% were missing race/ethnicity in the EHR. Most patients in this group (50.4%) were seen in California, while 23.2% were seen in Oregon and 26.4% in Washington. Patients whose preferred language was not English primarily used self-pay (29.7%), Medicare (26.2%), or Medicaid (20.2%), and encounters primarily took place in inpatient settings (30.5%), “other” settings (26.5%), or EDs (21.7%). The communities where patients whose preferred language was not English lived scored 0.53 and 0.62 on the SVI Socioeconomic and SVI Housing and Transportation domains, respectively.

**Fig. 1:**
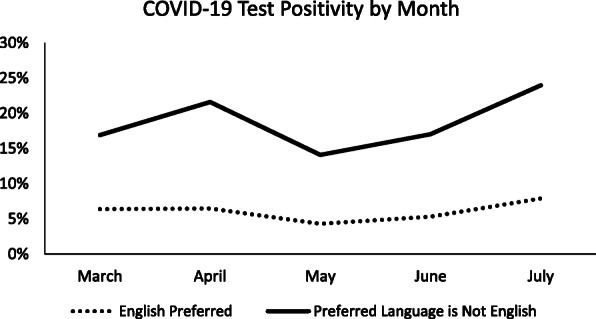
COVID-19 Test Positivity by Preferred Language (English or Non-English) Over Time in the Western U.S. in 2020. Line graph representing percent with a positive COVID-19 test by preferred language among the study population during the study window. The dotted line represents the percent with a COVID-19 positive test among those who preferred English. The solid line represents the percent with a COVID-19 positive test among those whose preferred language is not English

Figure 1 shows COVID-19 test positivity by month stratified by language. Positivity among patients whose preferred language was not English is consistently higher than that of English-speaking patients at all measured timepoints (between March and July 2020).

Language was significantly associated with COVID-19 test positivity (OR 3.07, *p* < 0.001), and this association remained significant after adjusting for age, race/ethnicity, and the two SVI domains (Table 2). After these adjustments, patients whose preferred language was not English had over twice the odds of testing positive for COVID-19 when compared to patient who preferred English (OR = 2.19, *p* < 0.001). Race/Ethnicity was also a significant driver of disparities in test positivity, with odds ratios ranging from 1.27 to 2.98 compared to a White reference group. Patients living in more vulnerable census tracts based on the SVI Housing and Transportation domain had higher odds of testing positive, but the socioeconomic SVI domain was not significantly associated with test positivity.

**Table 2:** Unadjusted univariate multilevel model and adjusted multivariate multilevel models for COVID-19 patients in the Western U.S. in 2020

	Model A			Model B			Model C		
	OR	95% CI	***p***-value	OR	95% CI	***p***-value	OR	95% CI	***p***-value
Language	3.07	2.92, 3.22	< 0.001	2.25	2.12, 2.39	**< 0.001**	2.19	2.06, 2.33	**< 0.001**
Age				1.00	1.00, 1.00	0.1322	1.00	1.00, 1.00	0.0505
Race/Ethnicity									
American Indian/Alaska Native				1.52	1.18, 1.97	**0.0013**	1.52	1.17, 1.96	**0.0015**
Asian				1.35	1.23, 1.48	**< 0.001**	1.33	1.21, 1.46	**< 0.001**
Black/African American				1.91	1.74, 2.10	**< 0.001**	1.82	1.65, 2.00	**< 0.001**
Hispanic				2.95	2.78, 3.12	**< 0.001**	2.87	2.71, 3.04	**< 0.001**
Multiracial				1.27	1.10, 1.46	**0.0008**	1.27	1.11, 1.46	**0.0007**
Native Hawaiian/Pacific Islander				3.06	2.53, 3.69	**< 0.001**	2.98	2.71, 3.04	**< 0.001**
Other				1.78	1.61, 1.95	**< 0.001**	1.76	1.59, 1.93	**< 0.001**
White				*ref*	–	–	*ref*	–	–
SVI Domain: Socioeconomic							1.09	0.98, 1.20	0.1001
SVI Domain: Housing and Transportation							1.19	1.09, 1.30	**0.0001**
R^2^			0.04			0.08			0.08

Because our outcome variable is COVID-19 test positivity, and not risk of infection, we also wanted to assess the impact of healthcare access in order to inform any conclusions we could draw from the main models. To do this, we included payer type and encounter setting in a sensitivity analysis; however, this had no impact in the overall risk of COVID-19 test positivity by preferred language (data not shown), so we did not retain these variables in the model.

The interaction between language and race/ethnicity was significant (*p* = 0.0004). Within each racial/ethnic category, patients whose preferred language was not English had higher odds of testing positive for COVID-19 than their counterparts who preferred English, with odds ratios ranging from 1.45 among Asian patients to 3.08 among Black/African American patients (Table 3). The interaction between language and state was also significant (*p* < 0.0001). Patients in California whose preferred language was not English had 84% higher odds of COVID-19 positivity than those who preferred English in California. Patients in Oregon and Washington whose preferred language was not English had close to three times the odds of COVID-19 positivity compared to English-speaking patients in each of those states (OR = 3.04 and 2.80, respectively; Table 3).

**Table 3:** Intersectionality between preferred language and race and preferred language and geography for COVID-19 patients in the Western U.S. in 2020

	Model D			Model E		
	OR^**a**^	95% CI	***p***-value	OR^**b**^	95% CI	***p***-value
**Race/Ethnicity**						
Asian	1.45	1.17, 1.79	**0.0006**			
Black/African American	3.08	2.12, 4.48	**< 0.001**			
Hispanic	2.3	2.12, 2.50	**< 0.001**			
Multiracial	2.44	1.53, 3.88	**0.0002**			
Native Hawaiian/Pacific Islander	2.67	1.64, 4.36	**< 0.001**			
Other	1.86	1.48, 2.34	**< 0.001**			
White	2.57	1.91, 2.67	**< 0.001**			
**State**						
California				1.84	1.69, 2.00	**< 0.001**
Oregon				3.04	2.57, 3.60	**< 0.0001**
Washington				2.8	2.52, 3.13	**< 0.001**

^a^OR comparing COVID-19 test positivity between people who prefer English (ref) and people whose preferred language is not English. Results stratified by race/ethnicity and adjusting for age, SVI Domain: Socioeconomic, SVI Domain: Housing/Transportation, and clustering of patients within states

^b^OR comparing COVID-19 test positivity between people who prefer English (ref) and people whose preferred language is not English stratified by state and adjusting for age, SVI Domain: Socioeconomic, and SVI Domain: Housing/Transportation, and clustering of patients within states

## Discussion

We found that patients whose preferred language was not English had three times the odds of testing postive for COVID-19, and that this relationship persisted after adjusting for race/ethnicity and social factors. We also found significant interaction between language and race/ethnicity and language and state, but overall the odds of COVID-19 test positivity remained greater for those whose preferred language was not English compared to people who preferred English within each race and state. This indicates language is a driver of the documented COVID-19 disparity regardless of race or state.

Recent research that investigated language phonology to understand whether characteristics of spoken language impacted COVID-19 transmission found no significant impact on COVID-19 transmission based on the language spoken [23]. This indicates that the disparity we observed in our study cannot be explained by characteristics of spoken language itself. Thus, other mechanism must explain the increased COVID-19 test positivity among people whose preferred language is not English in the context of where the predominant language is English.

One potential mechanism is that the population whose preferred language is not English have greater likelihood of having social factors (such as housing, job type, and transportation) associated with COVID-19 infection, which could explain disparities [4, 17–19]. We used dimensions of the SVI to consider the role of these factors in our analysis. Our descriptive analysis showed higher SVI in the socioeconomic and housing and transportation domains for patients whose preferred language was not English compared to those who preferred English. However, we found that even after adjusting for these SVI factors, language remained an independent predictor of increased risk of infection.

Another potential mechanism stems from extensive research that has demonstrated the many structural and systemic barriers to health faced by populations whose preferred language is not English [9, 10, 12–15]. One key barrier is English-centric public health communications [24]. COVID-19 risk mitigation relies heavily on high volume and rapidly evolving English-centric public health communications campaigns and recommendations [25, 26]. A recent research study on the effectiveness of COVID-19 communications among native English speakers demonstrated several key misconceptions on how to prevent acquisition of COVID-19, [27] indicating the difficulty of knowledge sharing even when there is language alignment. However, early studies of efforts to leverage emergency language services and management in the public health response demonstrated more limited COVID-19 spread [24].

The healthcare sector is another key system that supports educating the public about COVID-19 prevention and care – including access to COVID-19 vaccinations; however, people whose preferred language is not English face many barriers to accessing the U.S. healthcare system [10]. For non-English speakers, few providers are available from similar cultural backgrounds and/or who speak their native language [9]. Despite policy mandating that hospitals are required to provide professional language services, between a quarter to a third of hospitals do not have them in place, [28] leaving many hospital interactions to rely on family members or other ad hoc interpreters, which is known to be less effective and potentially disempowering for certain patients [9, 15]. Likewise, written materials in a patient’s preferred language are not consistently provided across healthcare encounters and settings [29]. There is also deep-rooted mistrust in healthcare due to historical injustice (especially in marginalized communities) and current discriminatory systems and practices [18, 30]. When people whose preferred language is not English seek healthcare in the U.S., they often do not receive all the needed information, leaving them with a poorer understanding of their health and care [10]. Research has shown that language-concordant providers, professional interpreters, and written materials in the patient’s native language can improve patient experience and outcomes [15, 31, 32]. Yet while there are some examples of how these strategies have been used in the treatment of COVID-19 – such as the formation of a Spanish-Language Care Group at a hospital in Massachusetts [19] – it is unclear from the published literature if and how these strategies are being used to communicate about COVID-19 prevention.

Additionally, cultural barriers can exist for people whose preferred language is not English. Health systems are recognizing culture as a determinant of health and are emphasizing the need for culturally competent training and care [30]. In recent years, there has been a growing focus on Community Health Workers – defined as frontline public health workers who are trusted members of and/or have close understanding of the community they serve [33] – and integrating them into health and healthcare teams to support culturally (and often linguistically) competent relationship building and trust to improve population health [33]. In addition, community-based organizations (CBOs) are often critical to filling gaps in culturally competent care and health systems emphasizing alignment with CBOs may lead to improved outcomes.

Across all three states on the West Coast, we found that the PSJH population whose preferred language was not English had increased odds of COVID-19 infection but with varying magnitudes of associations. California had the lowest OR and Washington had the highest; this is inversely proportional to the percent of patients preferring a language other than English in each state. The higher proportion of patients who speak languages other than English in California is unsurprising; California has the largest percentage of people in general in the U.S. who speak a language other than English [8]. California also has a higher proportion of healthcare providers who indicate they speak a non-English language, when compared to Oregon [34, 35]. Thus one potential explanation for the observed inverse association may be that states with a greater percent of patients preferring a language other than English may invest more in non-English interpreters and materials. Further research is needed to understand this relationship.

This study has several limitations to consider when interpreting the results. We are limited to the PSJH population in three states on the West Coast and in California we could only include hospitals using the same instance of EHR as Oregon and Washington. Findings may not be generalizable across the U.S. However, this study represents a diverse, multistate cohort that represents a large geographical area. This study was further limited to COVID-19 testing at PSJH, and patient demographics may look different at other testing sites. We adjusted for social risk factors; however, these values were based on census track and not available at an individual level, potentially leading to misclassification for specific patients. Health inequities beyond race/ethnicity, language, and social factors such gender identity and sexuality were not considered in the analysis – and these factors should be considered in future research. This study is also limited to exploring risk for COVID-19 test positivity, and not risk of COVID-19 infection overall. This limits our sample only to individuals who received a test, and this population may be different from those who did not get tested, especially in terms of access to healthcare. However, including healthcare access variables in our models had little impact on the overall results. Finally, this study did not examine outcomes after testing, therefore we do not know the effect of language on disease outcomes.

## Conclusions

We found that having a preferred language that is not English in the U.S. increases the risk of COVID-19 test positivity even when accounting for social factors and demographics including race. During a public health crisis, our public health systems have to be able to reach the entire population, including the large population of people in the U.S. whose preferred language is not English. There have been recent calls for a major linguistic shift in public health that embraces the linguistic and cultural diversity of our society. Taking action to better support the healthcare of people whose preferred language is not English in the U.S. is long overdue and especially urgent as we rollout COVID-19 vaccinations and continue to learn more about COVID-19 and how to best protect against infection and poor outcomes across all populations.

## Data Availability

The data used in this study was derived from PSJH electronic medical records and includes individual-level identifiers and protected health information. This is a closed database that is not available for public use.

## Abbreviations

PSJH: Providence St. Joseph Health
SVI: Social Vulnerability Index
EHR: Electronic Health Record
ED: Emergency Department
OR: Odds Ratio
CI: Confidence Interval
CBO: Community-based organization
PCR: Polymerase chain reaction
